# *Macrosolen bidoupensis* (Loranthaceae), a new species from Bidoup Nui Ba National Park, southern Vietnam

**DOI:** 10.3897/phytokeys.80.13338

**Published:** 2017-06-05

**Authors:** Shuichiro Tagane, Van Son Dang, Nguyen Van Ngoc, Hoang Thi Binh, Natsuki Komada, Jarearnsak Sae Wai, Akiyo Naiki, Hidetoshi Nagamasu, Hironori Toyama, Tetsukazu Yahara

**Affiliations:** 1 Center for Asian Conservation Ecology, Kyushu University, 744 Motooka, Fukuoka, 819-0395, Japan; 2 The VNM Herbarium, Institute of Tropical Biology, Vast, 85 Tran Quoc Toan Street, District 3, Ho Chi Minh City, Vietnam; 3 Department of Biology, Dalat University, 01 – Phu Dong Thien Vuong, Dalat, Vietnam; 4 Laboratory of Forest Resources and Society, Graduate School of Agriculture Kyoto University, Kitashirakawa-oiwakecho, Sakyo-ku, Kyoto, 606-8502, Japan; 5 Prince of Songkla University, Hat Yai, Thailand; 6 Institute of Ecology and Evolutionary Biology, National Taiwan University, Taipei, Taiwan; 7 Iriomote Station, Tropical Biosphere Research Center, University of the Ryukyus, 870 Uehara, Taketomi-cho, Yaeyama-gun, Okinawa, 907-1541, Japan; 8 The Kyoto University Museum, Kyoto University, Yoshida Honmachi, Sakyo-ku, Kyoto, 606-8501, Japan

**Keywords:** Bidoup Nui Ba National Park, DNA barcoding, Loranthaceae, *Macrosolen*, new species, Vietnam

## Abstract

*Macrosolen bidoupensis* Tagane & V.S.Dang, **sp. nov.** (Loranthaceae) is newly described from Bidoup Nui Ba National Park in Lam Dong Province, southern Vietnam. The new species is characterized by small broadly elliptic to circular leaves, sessile to short petioles, slightly cordate to rounded leaf bases, 4–5 pairs of lateral veins and a basally green corolla tube. An illustration, a summary of DNA barcoding of the plastid genes *rbcL* and *matK*, and a key to the species of *Macrosolen* in Vietnam are provided.

## Introduction

*Macrosolen* (Blume) Rchb. is a small parasitic shrub in the family Loranthaceae. The genus is characterized by being totally glabrous with 6-merous flowers subtended by one bract and two bracteoles (often connate), reflexed corolla lobes at anthesis, and 4-locular anthers. The genus comprises ca. 40 species widely distributed in tropical South and Southeast Asia. In Vietnam, it has been recorded by de Loureiro (1790, as *Loranthus*), Lecomte (1915, as *Elytranthe*), Danser (1938) and Hô (2003), and recently a taxonomic revision of Loranthaceae in the country by Han (2014) enumerated seven species of *Macrosolen*: *M. annamicus* Danser, *M. avenis* Danser, *M. bibracteolatus* (Hance) Danser, *M. cochinchinensis* (Lour.) Tiegh., *M. dianthus* Danser, *M. robinsonii* (Gamble) Danser and *M. tricolor* (Lecomte) Danser.

During botanical surveys in Bidoup Nui Ba National Park, Lam Dong Province, southern Vietnam in 2016, we encountered an undescribed species of *Macrosolen*. We here describe this new species as *Macrosolen bidoupensis* Tagane & V.S.Dang, and provide illustrations, and a key to the species of *Macrosolen* in Vietnam.

In addition to the morphological examination, DNA sequences are extremely helpful for delimiting species (Hebert and Gregory 2005, Dick and Webb 2012). We sequenced two DNA barcode regions of *rbcL* and *matK* following the recommendation of CBOL Plant Working Group (2009).

## Materials and methods

### Morphological observations

To verify the validity of the new species, we undertook morphological comparisons to closely related species based on herbarium specimens (ANDA, BKF, BO, BRUN, FU, HN, KYO, SAR, TNS, VNM), specimen images on the web (e.g. JSTOR Global Plants, http://plants.jstor.org/ and Muséum National d’Histoire Naturelle, http://www.mnhn.fr/), and consulting the relevant literature (e.g. Barlow 1995, 1997, 2002, Qiu and Gilbert 2003, Hô 2003, Han 2014).

### DNA barcoding

DNA amplification and sequencing of portions of the chloroplast genes *rbcL* and *matK* followed established protocols (Kress et al. 2009, Dunning and Savolainen 2010) as in Toyama et al. (2015).

## Taxonomy

### Macrosolen bidoupensis

Taxon classificationPlantaeSantalalesLoranthaceae

Tagane & V.S.Dang sp. nov.

urn:lsid:ipni.org:names:77163237-1

Figures 1 , 2

#### Diagnosis.

*Macrosolen bidoupensis* is similar to *Macrosolen tricolor* Danser of China, Laos and Vietnam in leaf shape and size and by possession of a 2-flowered umbel, but differs in having a sessile to very short petiole (up to 0.7 mm long in *M. bidoupensis* vs. 2–3 mm long in *M. tricolor*), more lateral veins (4 or 5 vs. 2 or 3 pairs), slightly cordate to rounded leaf bases (vs. cuneate) and a basally green corolla tube (vs. reddish around the basal 1/3). The leaf shape is also somewhat similar to *Macrosolen platyphyllus* Danser of Thailand, Peninsular Malaysia and Borneo, but easily distinguished in having much smaller leaves and flowers, and very short petioles.

**Figure 1.**
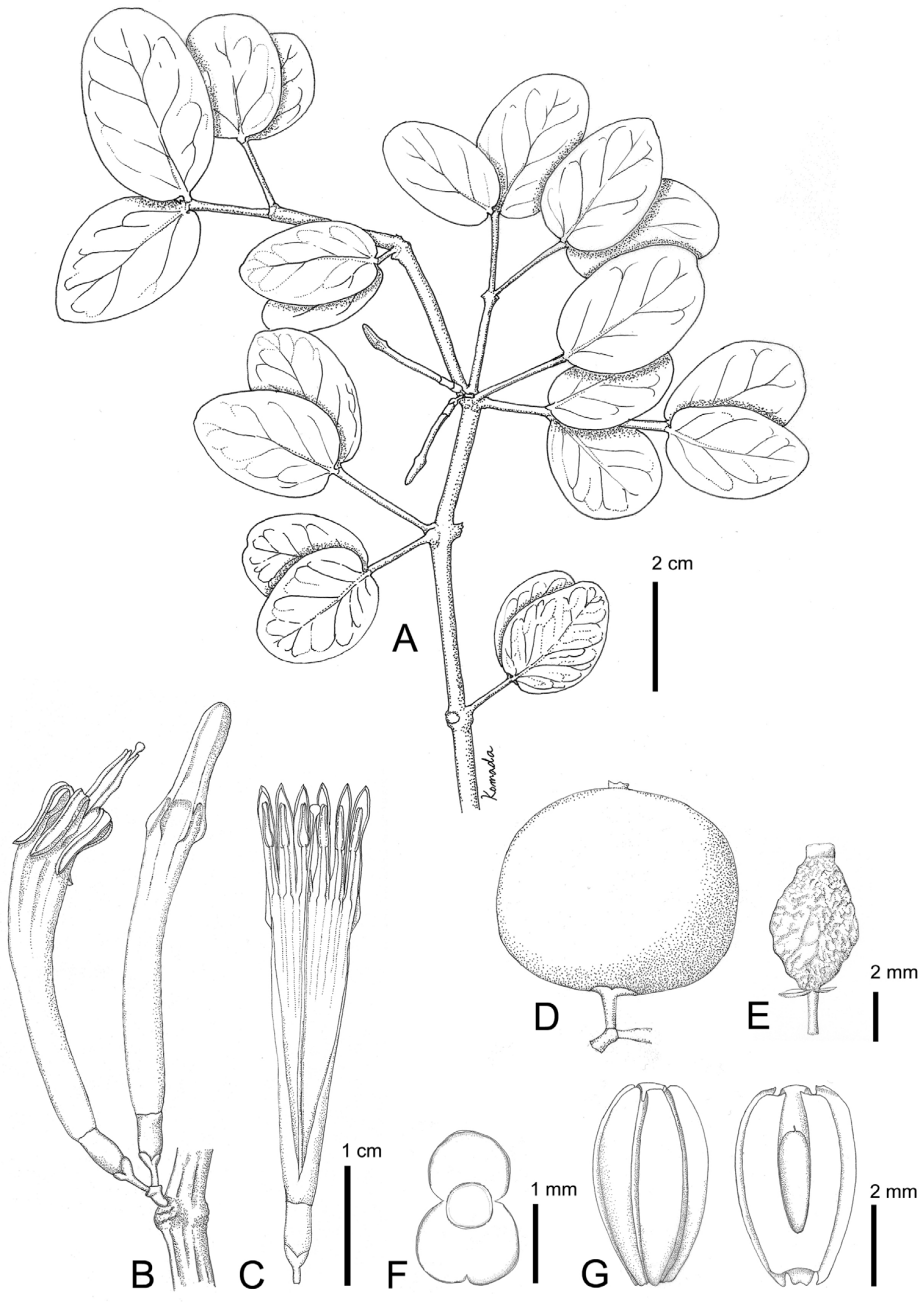
*Macrosolen bidoupensis* Tagane & V.S.Dang. **A** Branch with flower buds **B** 2-flowered inflorescence **C** Flower with corolla tube opened **D** Fresh fruit **E** Dried fruit **F** Seed **G** seed (left) and longitudinal section of seed (right). Materials: *Tagane et al. V4083* (KYO). Drawn by N. Komada.

**Figure 2.**
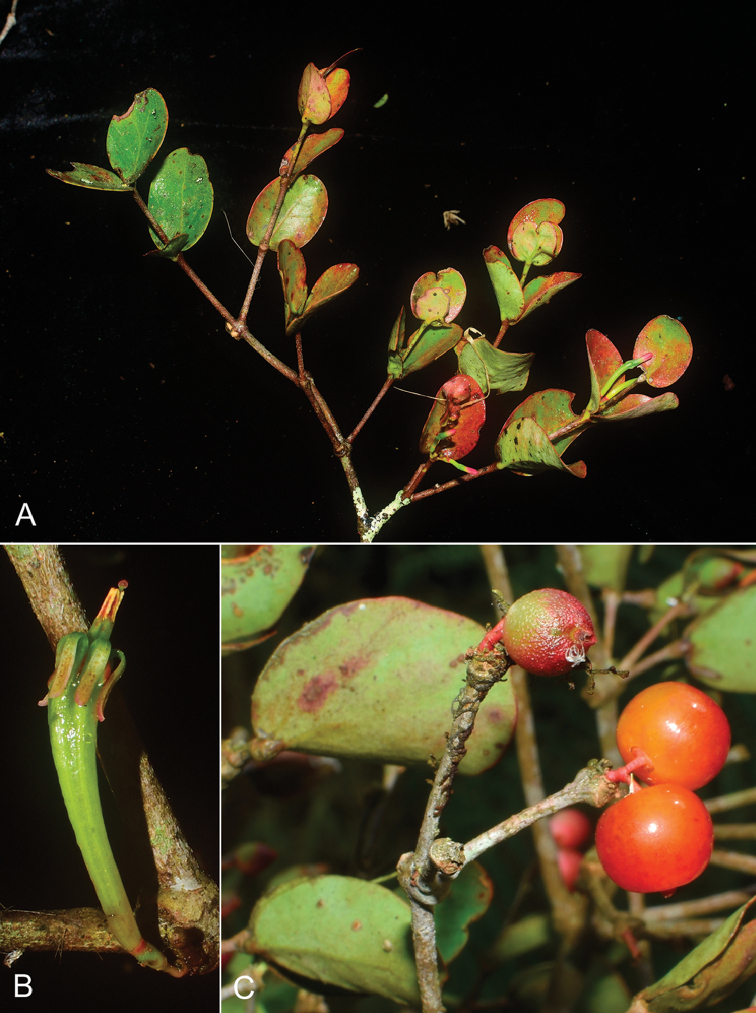
*Macrosolen bidoupensis* Tagane & V.S.Dang. **A** Branch with flower buds **B** Flower **C** Fruits. Photos by *Tagane et al. V4083*.

#### Type.

VIETNAM. Lam Dong Province, Bidoup Nui Ba National Park, in lower montane evergreen forest, 12°10'34.7"N, 108°41'08.4"E, alt. 1533 m, 21 Feb. 2016, with flowers, *Tagane S., Nagamasu H., Naiki A., Dang V. Son, Ngyuen V. Ngoc, Binh T. Hoang & Wai J. V4083* (holotype-KYO!, fl. spirit collection; isotypes NTU!, the herbarium of Bidoup Nui Ba National Park!, VNM!).

#### Description.

Hemi-parasitic shrub, 25–40 cm tall, totally glabrous. Branches terete, grayish to grayish brown, lenticellate. Leaves opposite; blade elliptic, broadly elliptic, broadly ovate-elliptic, or circular, 1.2–4.8 × 1.2–3.5 cm, coriaceous, apex broadly obtuse to rounded, base slightly cordate to rounded, margin entire, grayish green and slightly lustrous adaxially, dull olive abaxially, midrib prominent at basal 1/3 to 1/2 abaxially, secondary veins 4 or 5 pairs, obscure or visible adaxially, obscure to faintly visible abaxially; petiole to 0.7 mm long. Inflorescences axillary, sometimes at older leafless nodes, 2-flowered umbels; peduncle ca. 0.9 mm long. Pedicel 1.1–1.5 mm long; central bract suborbicular, ca. 1 mm long, bracteoles suborbicular, connate, ca. 1 mm long; mature buds 2.8–3.2 cm long; calyx limb (calyculus) annular, ca. 0.7 mm long; corolla green tinged with red at top of tube and lobes, symmetrically 6-winged at about two thirds the length, tube 2.2–2.4 cm long in anthesis, gradually dilated, inflated, slightly curved, lobes lanceolate, 6–8 mm long, reflexed in anthesis; stamens 6, anthers ca. 2.3 mm long, filaments basally adnate to corolla tube, free part 2.3–3.3 mm long; ovary 2.8–3.3 mm long; style 2.8–3.2 cm long, stigma capitate. Berry reddish orange, subglobose to slightly depressed-barrel-shaped, ca. 8 mm high, ca. 6 mm wide, ovoid when dry, apex beaked by calyx limb. Seed 1, ellipsoidal, ca. 5 mm high, ca. 3 mm wide, longitudinally 6-grooved. The measurements of the flower characters are derived from the spirit collection.

#### Other specimen examined in Vietnam.

Bidoup Nui Ba National Park; in lower montane evergreen forest, 12°10'34.9"N, 108°41'04.4"E, alt. 1533 m, 22 Feb. 2016, with fruits, *Tagane et al.V4169* (FU, NTU, the herbarium of Bidoup Nui Ba National Park, VNM).

#### Phenology.

Flowering and fruiting specimens were collected in February.

#### Distribution and habitat.

Vietnam (so far known only from Bidoup Nui Ba National Park, Lam Dong Province). In lower montane evergreen forest, ca. 1500 m altitude.

#### GenBank accession no.

*Tagane et al. V4083*: LC259010 (*rbcL*), LC259011 (*matK*). The partial *rbcL* sequence of *M. bidoupensis* was identical to *M. tricolor* (GenBank accession no. HQ317771) of the total 517 bp and differed at 3 bases of the 518 total from *M. cochinchinensis* (KP094775 and HQ317768). The partial *matK* sequence of *M. bidoupensis* differed at 23 bases from *M. cochinchinensis* (EU544439) of the 813 total.

#### Etymology.

The specific epithet *bidoupensis* is derived from its type locality.

#### Conservation status.

Least Concern (LC). Bidoup Nui Ba National Park is located on Central highland of Vietnam. It covers ca. 700.38 km^2^ and is the core zone of the Langbiang biosphere reserve (Bidoup-Nui Ba National Park 2016). *Macrosolen bidoupensis* is commonly found in the lower montane evergreen forest in the national park. Since the forest is widely seen and well-protected, we suggest this status as LC according to IUCN (2012).

#### Note.

A similar species, *Macrosolen tricolor* is known from lower elevations (below 100 m, Qiu and Gilbert 2003), and the two species do not occur sympatrically.

### Key to the species of *Macrosolen* in Vietnam

**Table:** 

1	Leaf blade 1.2–5.5 × 1.2–3.5 cm, apex rounded to broadly obtuse	**2**
–	Leaf blade (3–)4–15 × (1.2–)1.5–7 cm, apex acute to acuminate, or obtuse	**3**
2	Petiole to 0.7 mm long; lateral veins 4 or 5 pairs	***M. bidoupensis***
–	Petiole 2–3 mm long; lateral veins 2 or 3 pairs	***M. tricolor***
3	Mature flower bud 1–1.5(–2.3) cm long	**4**
–	Mature flower bud 3–8.5 cm long	**5**
4	Petiole (1–)5–10 mm long; inflorescences racemose, (2–)4–8-flowered; peduncle (5–)15–20 mm long	***M. cochinchinensis***
–	Petiole 2–4 mm long; inflorescences umbellate, 2- or 3-flowered; peduncle 1–2.5 mm long	***M. robinsonii***
5	Flowers subsessile, pedicel less than 1 mm long; bract 2–3 mm long; anther 5–8 mm long	***M. dianthus***
–	Flowers distinctly pedicellate, pedicel 2–6 mm long; bract 1–1.5 mm long; anther 2–5 mm long	**6**
6	Mature flower bud more than 4.5 cm long; anthers 4–6 mm long	***M. annamicus***
–	Mature flower bud 3–4 cm long; anthers 2–3 mm long	**7**
7	Corolla tube curved near the middle, asymmetrically winged	***M. avenis***
–	Corolla tube curved above the middle, symmetrically winged	***M. bibracteolatus***

## Supplementary Material

XML Treatment for Macrosolen bidoupensis
